# A whole genomic scan to detect selection signatures between Berkshire and Korean native pig breeds

**DOI:** 10.1186/2055-0391-56-23

**Published:** 2014-11-06

**Authors:** Zewdu Edea, Kwan-Suk Kim

**Affiliations:** Department of Animal Science, Chungbuk National University, Cheongju, 361-763 Korea

**Keywords:** Korean native pig, Genome-wide, SNP, Selection signature

## Abstract

**Background:**

Scanning of the genome for selection signatures between breeds may play important role in understanding the underlie causes for observable phenotypic variations. The discovery of high density single nucleotide polymorphisms (SNPs) provide a useful starting point to perform genome–wide scan in pig populations in order to identify loci/candidate genes underlie phenotypic variation in pig breeds and facilitate genetic improvement programs. However, prior to this study genomic region under selection in commercially selected Berkshire and Korean native pig breeds has never been detected using high density SNP markers. To this end, we have genotyped 45 animals using Porcine SNP60 chip to detect selection signatures in the genome of the two breeds by using the *F*_*ST*_ approach.

**Results:**

In the comparison of Berkshire and KNP breeds using the FDIST approach, a total of 1108 outlier loci (3.48%) were significantly different from zero at 99% confidence level with 870 of the outlier SNPs displaying high level of genetic differentiation (*F*_*ST*_ ≥0.490). The identified candidate genes were involved in a wide array of biological processes and molecular functions. Results revealed that 19 candidate genes were enriched in phosphate metabolism (GO: 0006796; *ADCK1, ACYP1, CAMK2D, CDK13, CDK13, ERN1, GALK2, INPP1*; *MAK, MAP2K5, MAP3K1, MAPK14, P14KB, PIK3C3, PRKC1, PTPRK, RNASEL, THBS1, BRAF, VRK1*). We have identified a set of candidate genes under selection and have known to be involved in growth, size and pork quality (*CART*, *AGL*, *CF7L2, MAP2K5, DLK1, GLI3, CA3* and *MC3R)*, ear morphology and size (H*MGA2* and *SOX5)* stress response (*ATF2,* MSRB3*, TMTC3* and *SCAF8)* and immune response ( *HCST* and *RYR1*).

**Conclusions:**

Some of the genes may be used to facilitate genetic improvement programs. Our results also provide insights for better understanding of the process and influence of breed development on the pattern of genetic variations.

**Electronic supplementary material:**

The online version of this article (doi:10.1186/2055-0391-56-23) contains supplementary material, which is available to authorized users.

## Background

Pigs have long been of great economic importance to many farmers in the world. Molecular evidence supports independent domestication of pig in Asia and Europe from wild boar sub-species [1, 2]. As compared to their wild ancestor, domestic pig breeds display a wide range of phenotypic variations that have been manipulated and shaped during the course of domestication and breed development for a wide range of traits. Some pig breeds, particularly commercial breeds have been intensively selection for better growth, meat quality and fertility traits may have resulted in loss of genetic diversity. To the contrast, most traditional breeds are reared by smallholder farmers and less subjected to selection pressure and harbor higher genetic diversity for adaptation under marginal environments.

The superiority of some commercial pig breeds for growth and carcass traits over traditional breeds, have led them to be the breed of choice and their continuous utilization in improvement of native populations through crossbreeding. In Asia, it has been known that commercial breeds have contributed to the genetic pool of most indigenous breeds [3]. Likewise, for the last two decades, western pig breeds have been imported into the Korea peninsula and crossed with the Korean native pigs (KNP) in order to improve growth and carcass related traits [4]. As a result the number of Korean native pigs decreased noticeably following the introduction of improved breeds. Although commercial breeds are superior in terms of growth and feed efficiency traits, the Korean native pig harbors unique genetic material for product quality and better adaptation to low management levels [5]. Despite the indiscriminate crossbreeding, little is known regarding the genome composition difference between the Korean native and European (Berkshire) pig breeds.

There is a growing interest in spotting genomic regions or genes that have been under selection. *F*_*ST*_ statistic is among the most widely used measures to identify genomic regions or loci that display high differentiation between populations [6]. Genomic regions or loci that show significantly high *F*_*ST*_ values compared with neutral loci offer evidence for positive selection. Until recently, there has been little success of detecting genomic regions under selection in livestock species attributed to lack of high density molecular markers. However, through the advancement of high-throughput sequencing technology, thousands of single nucleotide polymorphisms have been discovered and open opportunities to facilitate and transform livestock genetic improvement programs. In pig, several thousands of SNPs spinning the whole genome has been discovered using next generation technologies [7]. The availability and discovery of such large number of SNPs provide a useful starting point to perform genome–wide scan in pig populations in order to identify candidate genes underlining phenotypic variations between breeds. However, prior to this study genomic region under selection in commercially selected pig breed like Berkshire and Korean native pig has never been detected using high density SNP markers. Scanning of the genome for selection signature between highly selected and traditional breeds may play important role in identifying genes underlying for phenotypic variation. In addition, it can be used to facilitate genetic improvement and conservation programs. To this end, we have genotyped 45 animals are using Porcine SNP 60 BeadChip to identify loci variants showing directional selection in comparing European (Berkshire) and Korean native pig breeds using the *F*_*ST*_ approach.

## Methods

### Pig breeds, sampling and genotyping

Samples were collected from unrelated Berkshire (n = 29) and from Korea native pig (KNP, n = 16) breed. Briefly, Korean native pig was phenotypically discriminated as long black coarse hairs, long straight noses, greatly protruded mouth and straightly upright ears. The breed is known for its high prolificacy, better meat quality (high redness and intramuscular fat [5] and strong adaptability under low management conditions, but showed a slower growth rate, small adult body weight, smaller litter sizes, and lower carcass yield [8]. On the other hand, Berkshire pig breed is characterized by medium to large body size, fast growth rate, early maturing, and large litter size, medium and erect ears.

DNA samples of Korean native pig were obtained from National Institute of Animal Science (NIAS) and that of Berkshire were obtained from Dasan Breeding Farm in Korea. Sample collection procedures were approved by the National Institute of Animal Science (NIAS). During sample collection animals were treated humanely. All animals were genotyped performed using the Illumina Pocrine SNP60 BeadChip [9]. Common monomorphic SNPs for all of the breeds were discarded from further analyses. SNPs were filtered with criteria of call rate (≥90%), minor allele frequency (MAF ≥5%) and Hardy-Weinberg equilibrium (HWE ≥0.001). Thus finally about 31,755 SNPs were considered for the study.

### Statistical analysis

#### Genetic variations

Genetic diversity was assessed for each breed by calculating observed and expected heterozygosities using Arlequin software [10]. Principal component analysis was performed to illustrate the pattern of individual clustering using SNP and Variation Suite version 7 [11]. PCA assigns individuals to their population of origin using a common clustering algorithm Patterson *et al.* [12]. In the principal component analysis, the first principal component (PC1) accounts for the greater variation followed by principal component (PC2).

#### Detection of outlier loci or signature of selection

Detection of outlier loci was based on calculation of fixation index (*F*_*ST*_*)* at different significance levels as a measure of genetic differentiation for each locus between Berkshire and KNP following the FDIST approach proposed by [13] as implemented in Arlequin software [10]. Briefly, the FDIST program calculate genetic differentiation index (*F*_*ST*_) for each loci and then uses coalescent simulation to generate the null distribution of *F*_*ST*_ values based on the infinite island model [13]. Within this framework, we ran 20,000 coalescent simulations to obtain the P − values of locus-specific *F*_*ST*_ conditioned on observed levels of heterozygosity with default settings. This method provides evidence for divergent selection by looking for outlier loci with *FST* values higher than expected, controlling for heterozygosity. The corresponding candidate genes for outlier SNPs (P <0.01) were annotated with the pig genome analysis data repository [14].

#### Biological process and molecular functional analyses of the candidate genes under selection

To known the biological process and molecular functional of each candidate genes, we assessed their Gene Ontology (GO) and classification using a web-based Database for Annotation, Visualization, and Integrated Discovery (DAVID) tools [15]. Furthermore, enrichment analysis was performed to identify biological processes and molecular functions over-represented by Fisher Exact test (EASE score). Any GO terms that have a larger than expected subset of selected genes were considered over-represented and gave insight into the functional characteristics of the annotated genes.

#### Haplotype blocks detection

To investigate whether any of the significantly differentiated loci or genes (P <0.01) are in strong linkage disequilibrium, we further analyzed LD and haplotype blocks for the two breeds following the [16] method using the SNP and Variation Suite version 7 [11]. According to this method, SNP pairs to be in strong linkage disequilibrium (LD) if the one-sided upper 95% confidence bound on D’ is.0.98 (that is, consistent with no historical recombination) and the lower bound is above 0.7.

## Results

### Genetic diversity and population structure

**Figure 1:**
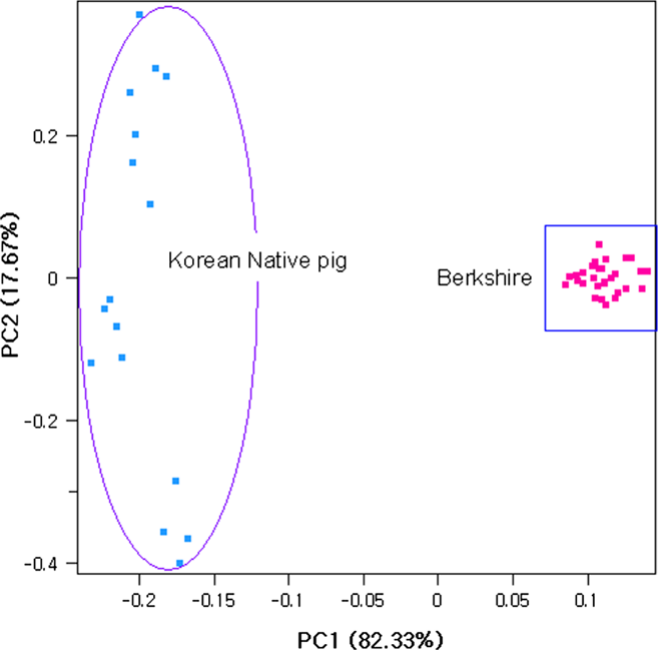
**Individual animal clustering on the basis of principal component analysis.**

The average observed heterozygosity was 0.321 ± 0.171 in Berkshire and 0.326 ± 0.173 in Korean native pig, whereas the expected heterozygosity was found to be 0.319 ± 0.156 and 0.336 ± 0.153 for Berkshire and Korean native pig, respectively. The average within − breed fixation index (*F*_*IS*_) was shown deficiency of heterozygosity (0.029) in Korean native pig whereas it was negative (−0.008) in Berkshire. To illustrate the pattern of individual animals clustering, we performed principal component analyses (PCA). Principal component one (PC1) and principal component 2 (PC2) accounted for 82.33% and 17.67% of the total variance, respectively (Figure 1) and clearly separated individuals according to their breed group.

### Genetic differentiation, outlier loci and candidate genes under selection

**Figure 2:**
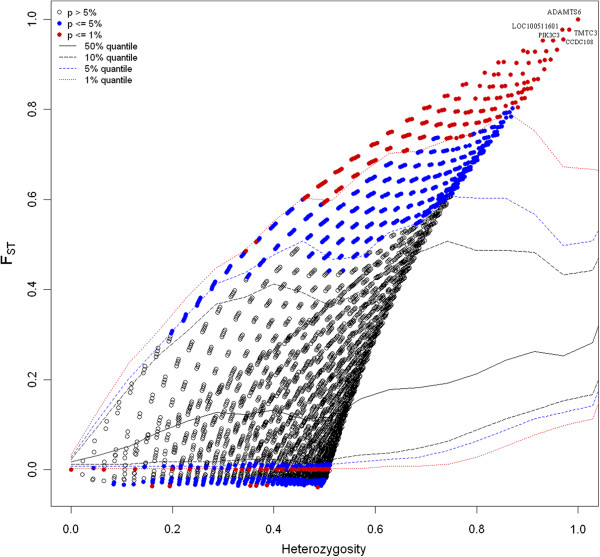
**Joint distribution of** ***F***_***ST***_ **and heterozygosity based on the 31755 SNPs analyzed for Berkshire and Korean native pig breed comparison.** Loci significant at 5% and 1% levels are indicated by blue and red circles, respectively, as estimated using FDIST approach of [12]. The red, blue, solid and broken lines represent the 1%, 5%, 10% and 50% quintiles, respectively, indicating the point at which 99%, 95% and 50% of the data fall above that value, respectively.

In this study, we are primarily intended to identify outlier loci in the comparison of two pig breeds (Berkshire and Korean native pigs). Level of differentiations between the breeds was measured by fixation indices. The overall *F*_*ST*_ was 0.157 with about 29% (9127) of the loci having an *F*_*ST*_ value below zero or equal to zero. The highest genetic differentiation between the two breeds was observed on chromosome 16 where 7 SNPs or loci (rs81228734, rs81458940, rs81459172, rs81459185, rs81459183, rs81297918 and rs81459195) displayed an *F*_*ST*_ value of 1. In the comparison of Berkshire and KNP breeds, using the FDIST approach indicated that a total of 1108 loci (3.48%) were significantly different from zero at 99% confidence level with 870 of the outlier SNPs displaying a high level of genetic differentiation (*F*_*ST*_ ≥ 0.48) (see Additional file 1) and revealing that the loci are potentially under directional selection. The distribution of *F*_*ST*_ as a function of expected heterozygosity based on the 31755 loci is presented in Figure 2. The *F*_*ST*_ value plot by chromosome is given in Additional file 2.

### Haplotype blocks

The distribution of haplotype blocks in Korean native pig and Berkshire are shown in Additional file 1: Table S1. The overall the distribution of haplotype blocks was higher in Korean native pig where a total of 76 variable size blocks was identified. In the contrast, in the Berkshire only 32 haplotype blocks were detected. The number of haplotype blocks detected in the Korean native pig population ranged from 10 in chromosome 5 to none in chromosome 6 and 9. In contrast, the Berkshire population had the highest number of haplotype blocks (7) in chromosome 7 and no haplotype block was identified for pairs of SNPs in chromosome 10, 11, 14, 17 and 18. The size of each block varied from 5.716 kb to 158.824 kb in KNP and ranged from 11. 916 kb to 158.270 kb in Berkshire samples.

The first block, in chromosome 1 in Korean native pig covered 116.539 kb and contained IGF2R gene whereas the second block of the same chromosome spanned about 55.54 kb and covers SCAF8 gene. Two blocks in chromosome 5 (first and second blocks) which spanned 51.785 kb and 137.242 kb, respectively encompassed WIFI candidate gene which is known to be associated with bone development. Similarly, the first block in chromosome 5 spanned about 51.785 kb and encompassed important gene (MSRB3) which is known to be related to cold and heat stresses. In chromosome 17, the third haplotype block covers MC3R gene previously known to be associated with body weight, adipose mass and feed conversion efficiency. The two breeds shared 12 common haplotye blocks. Of these blocks, block 69 in Korean native pig and block 28 in Berkshire spanned 158.27 kb and includes HOXA10 gene that play an important role in morphogenesis (Additional file 3). We compared the pattern of *F*_*ST*_ and haplotype blocks distribution. The highest genetic differentiation (*F*_*ST*_ =1.00) values were observed for SNPs positioned within chromosome 16, but there was no any halotype block detected for these particular SNPs. Common for both breeds, the largest haplotype blocks were detected on chromosome 15 (87,700,078 - 87,163,500) with average *F*_*ST*_ value of 0.82, while the smallest haplotype (12.848 kb) was detected for chromosome 13 with these SNPs displaying an average genetic differentiation of 0.00.

### Gene ontology (GO) term analyses of the candidate genes under selection

The candidate genes were analyzed for their gene ontology (GO) and tested for enrichment based on a Fisher Exact test to identify biological process and molecular functions most pertinent by our genes list. The biological process and molecular functions of the candidate gene are present in Additional files 3 and 4, respectively. The identified candidate genes involved in a wide array of biological processes and molecular functions. Results revealed that 19 candidate genes were enriched in phosphate (GO: 0006796) and phosphorus metabolisms (GO: 0006793). GO term enriched in ion transport contained 16 candidate genes, while 5 (*GLI3, CHD7, FBN2, HOXA10* and *NR2F2*) and 4 (*GLI3, CHD7, FBN2* and *HOXA10*) candidate genes were involved in limb development (GO: 0060173) and morphogenesis (GO: 0035108), respectively. In addition, our functional analysis demonstrated that higher numbers of the candidate genes with significant enrichment had molecular functions related to ion binding (48). We also detected a significant encirclement for candidate genes involved in nucleotide and nucleoside bindings. Molecular function enriched with ATP bindings contained 23 candidate genes (*ATP2A3, ADCK1, CAMK2D, CHD7, CDKL3, DGKB, ERN1, GALK2, KIF6, MAK, MAGI3, MAP2K5, MAP3K1, MAP3K14, MYO1B, PI4KB, PIK3C3, PRKCI, RNASEL, LOC392335, LOC441420, MYO5B, UBE2W, BRAF* and *VRK1*) under selection. About 15 and 12 potential candidate genes were involved in protein kinase and protein serine/threonine kinase activity, respectively.

## Discussion

The post-genomics era have opened opportunity in the scanning of whole for election signature in most commercial livestock species. Selection signatures may be used to identify genes or chromosomal regions that are possible targets of positive selection. The detection of selection signatures for local adaptation and phenotypic variations in Korean and western pig breeds is yet lacking. In this study, we compared two phenotypically distinct pig breeds in order to identify a sub-set loci significantly differentiated by employing an *F*_*ST*_ test in porcine 60 K SNP chip. We detected 1108 outlier loci (p <0.01) showing signature of selection and some of associated with genes known to be associated with production traits, ear size and morphology and diseases.

Growth and carcass qualities are important traits influencing the pig industry and these traits have been received considerable attention in breed improvement programs. Modern pigs have selected for lower levels of fat and fast lean growth [17]. In support with these facts, we identified potential candidate genes associated with growth, fat composition and feed conversion efficiency. Some of the genes include: *CART*, *AGL*, *CF7L2, MAP2K5, DLK1* and *MC3R.* For example study by [18] revealed that chromosomal region harboring the *CART* gene is a promising QTL in pig production traits (abdominal fat, weight and back fat thickness). In addition, this gene plays a crucial role in a variety of physiological processes, including food intake and body weight regulation [19]. Recent genome-wide association studies (GWAS) found that genetic polymorphisms in the *AGL* gene has shown to be association with growth and carcass traits in the crossbred population of Landrace and Jeju (Korea) Black pig [20]. We also detected signature of selection at *TCF7L2* loci which found to be associated with fat deposition traits in pigs [17]. This gene is known to locate on chromosome 14 where chromosome − wide significant trait loci for last ribs back fat and carcass weight were detected in Berkshire and Yorkshire crosses population [21].

Another important candidate gene under selection is *MAP2K5. MAP2K5* associated with body mass index and obesity in human [22]. Furthermore this gene is a component of the MAPK-family intracellular signaling pathways, responding to extracellular growth factors 2 (*IGF2*) [23]. Interestingly, we detected selection signatures at *DLK1* which is one of among imprinted genes in the callipyge locus (*CLPG*) region and associated with fat deposition, lean muscle mass and prenatal and postnatal growth rates in pigs [24]. In the comparison of the small sized KNP against the medium sized Berkshire, we identified selection footprints in *HMGA2* and *CA3* genes which were previously known to be associated with meat quality traits [25].

We have additionally identified two candidate genes, *GLI3* and *MC3R,* that known to influence body weight and growth traits*. GLI3* is associated with growth traits [26]. Melanocortin-3 receptor (*MC3R*) was previously reported to affect adipose mass in mice [27]. This gene is also associated with feed conversion and body weight in broiler [28] and with body weight in cattle [29]. KNP grow slower compared to the faster growing ability of European commercial pig breed (Berkshire). These genes may be involved for observable phenotypic variations in terms of growth traits in the two breeds and could serve as a molecular marker in the breeding programs. Therefore, our study provides evidence that these candidate genes detected here are likely under selection for better carcass quality traits and may be used for marker-assisted selection in beef cattle breeding program.

Among the potential candidate genes displaying signature of selection signature are *ATF2, MSRB3, TMTC3* and *SCAF8* genes which were involved in stress responses [30, 31]. Particularly the *MSRB3* gene is known to play a key role in protection mechanisms against cold and heat stresses [32]. Considering the extreme environmental temperature where KNP is originated and developed, this gene is likely under selection for cold resistance.

Breed difference for disease resistance quite obvious between improved and native breeds. KNP is known to have adaptability under low management systems. We identified genes known to playing physiological functions of either inhabiting or activating immune response. These include pig immune receptor (*HCST* or *DAP1*0) which was detected predominantly in lymphohematopoietic tissues [33]. Studies in humans and mice demonstrated that *DAP10* and *DAP12* can either activate or inhibit immune responses [34] implying that they play an important role in innate immune responses. Malignant hyperthermia (MH) causes major economic losses in the swine industry. Interestingly, here we detected signature of selection in *RYR1* gene which is an essential gene in swine. A single point mutation in the *RYR1* gene was found to be correlated with MH in breeds of swine [35].

Pigs have undergone morphological evolution through the course of domestication and breed development. For instance, strong selection signatures have been detected in loci harboring quantitative trait loci that explain morphological changes in the domestic pig [36]. In line with this evidence, our GO classification analysis indicated that some of the candidate genes under selection were known to be associated with limb development and morphogenesis. Among others, ear size and morphology are important conformation characteristics for breed discrimination. In this study we detected to two potential candidate genes (H*MGA2* and *SOX5*) known to have a key role in affecting ear size and morphology. *HMGA2-LPP* fusion protein promotes chondrogenesis [37]. More interestingly, studies revealed that *HMGA2*-deficient mice develop smaller ears [38] and in dogs, it may be involved in differences in the size and type of ears [39]. Furthermore, *SOX5* plays a role in chondrogenesis [40]. Considering the distinct variations in ear morphology and size displayed by the study populations, the detected genes are potentially under selection for the observable differences. Considerable variation was observed regarding to the number and distribution of haplotype blocks in the two breeds. The relatively higher number of haplotype blocks detected in the KNP population is consistence with the demographic history of the breed [4]. The bottle neck associated with reduction in KNP population may lead to greater LD.

Modern pig breeds have been selected for reproduction traits. KNP is known for its high prolificacy and here we identified some potential genes having role in reproduction or fertility. Possible role of phospholipase B in sperm maturation and activation was investigated in guinea pig [41]. *KIF6* or *KRP3* gene has involved in spermatid maturation mediated by possible interaction with the Ran GTPase [42]. Previously *PDE3A* is identified as the major cAMP-degrading PDE in the oocyte and regulates the resumption of meiosis [43].

## Conclusions

In this study we identified several candidate genes which have known associated with pork production (growth, size, and pork quality), morphology, stress and immune response. Some of the genes may be used to facilitate genetic improvement programs. Our results also provide insights for better understanding of the process and influence of breed development on the pattern of genetic variations. As the current annotation of pig genome is not conclusive, it is worth noting that many of the outlier loci or genes without GO terms may have relevant biological meanings and functions.

## Authors’ information

E.Z PhD student at Chungbuk National University and graduate fellow at ILRI; K.S.K professor of Animal Science at Chungbuk National University.

## Electronic supplementary material

Additional file 1: **Loci displaying selection signature at 99% confidence level and candidate genes in the comparison of Berkshire and Korean native pig breeds.** (XLS 306 KB)

Additional file 2: ***F***_***ST***_ **plot by chromosome in the comparison of Koran native and Berkshire pig breeds.** (XLS 508 KB)

Additional file 3: **Biological process of the candidate genes under selection based on gene ontology (GO) analysis.** (XLS 12 KB)

Additional file 4: **Molecular functions of the candidate genes under selection based on gene ontology (GO) analysis.** (XLS 13 KB)

## Abbreviations

DAVID: Database for Annotation, Visualization, and Integrated Discovery
DNA: Deoxyribonucleic acid
EASE: Expression analysis systematic explorer
*F*_*IS*_: Within − breed fixation index
*F*_*ST*_: Fixation index
GO: Gene ontology
ILRI: International Livestock Research Institute
HWE: Hardy-Weinberg equilibrium
KNP: Korean native pig
MAF: Minor allele frequency
NIAS: National Institute of Animal Science
PC: Principal component
PCA: Principal component analysis
SNP: Single nucleotide polymorphism.
